# Simple, One-Pot Method for Preparing Transparent Ethyl Cellulose Films with Good Mechanical Properties

**DOI:** 10.3390/polym14122399

**Published:** 2022-06-14

**Authors:** Gabrijela Horvat, Klara Žvab, Željko Knez, Zoran Novak

**Affiliations:** 1 Faculty of Chemistry and Chemical Engineering, University of Maribor, Smetanova ul.17, 2000 Maribor, Slovenia; gabrijela.horvat@um.si (G.H.); klara.zvab@um.si (K.Ž.); zeljko.knez@um.si (Ž.K.); 2 Faculty of Medicine, University of Maribor, Taborska Ulica 8, 2000 Maribor, Slovenia

**Keywords:** ethyl cellulose, film formation, plasticizers, food packaging

## Abstract

In this research, ethyl cellulose films were prepared by a simple, easy, controlled one-pot method using either ethanol or ethyl lactate as solvents, the films being formed at 6 °C. Titanium dioxide nanoparticles were incorporated to improve the oxygen transmission and water vapour transmission rates of the obtained films. This method used no plasticizers, and flexible materials with good mechanical properties were obtained. The resulting solvent-free and transparent ethyl cellulose films exhibited good mechanical properties and unique free-shapable properties. The obtained materials had similar properties to those reported in the literature, where plasticizers were incorporated into ethyl cellulose films with an elastic modulus of 528 MPa. Contact angles showed the hydrophobic nature of all the prepared materials, with contact angles between 80 and 108°. Micrographs showed the smooth surfaces of the prepared samples and porous intersections with honeycomb-like structures. The oxygen and water vapor transmission rates were the lowest for the ethyl cellulose films prepared in ethyl lactate, these being 615 cm^3^·m^−2^·day^−1^ and 7.8 gm^−2^·day^−1^, respectively, showing that the films have promise for food packaging applications.

## 1. Introduction

Food packaging is mainly made from different polymers, most of which are plastics. Such materials exist in the form of films or unique shapes desired by the industry. Plastic packaging is made of low-cost materials that are flexible and have good chemical stability. However, this type of food packaging is highly polluting and, despite the many recycling processes, most plastic packaging is disposed of into the environment, making it a major source of pollution. Due to the nondegradability of conventional plastic materials, there is an increased demand for non-petroleum-based materials that can degrade over time [1,2]. Such biodegradable materials could be prepared from renewable resources or environmentally friendly and completely green materials, such as polysaccharides. The drawback of the latter is that, often, polysaccharides are water-soluble, their final products (such as films) being degraded in water [3]. 

Ethyl cellulose (EC) is a common polysaccharide with hydrophobic properties and is considered a biodegradable polymer. This polymer is already extensively used for films, thickening agents, and adhesives in food and in the pharmaceutical and paper industries [4,5]. EC is stable when exposed to light, heat, oxygen, water, or chemicals. This polymer is a suitable choice for oxygen-scavenging applications, e.g., in food packaging applications. It is compatible with other polymers and plasticizers and can therefore be used to make waterproof films [6,7,8]. As it is heat-resistant, it does not produce toxic substances upon exposure to heat, unlike polyvinylchloride (PVC). The formation of the thin film is as simple as dissolving the EC in ethanol, followed by the addition of plasticizers. The drawback of plasticizers is that the EC film can change the transparency rate or become non-transparent, and the glass transition temperature can decrease [9]. However, plasticizers, such as dibuthyl sebacate or diethyl phthalate (Hyppölä et al., 1996), improve films’ flexibility and mechanical properties [10]. To achieve the desired mechanical properties, the amount of plasticizers used is usually high, up to 15–20 wt% [11]. Another drawback of plasticizers is that, due to their low molecular weight, they can leach from EC films so that, over time, the films become rigid [12]. Therefore, there is a desire to eliminate those materials from the production process while preserving the good mechanical properties. 

Besides good mechanical properties and degradability, food packaging materials also have other criteria to meet before final applications. Oxygen transmission rate (OTR) and water vapor transmission rate (WVTR) are important parameters in the evaluation of materials’ suitability for food packaging. Biopolymer films are still far behind the conventional plastic materials in terms of WVTR [13,14], but OTR values are also high in some traditional plastics, such as polypropylene (PP) [15]. Incorporating nanoparticles could enhance the barrier properties of packaging films; however, this also affects the mechanical properties of films [16]. Lowering both OTR and WVTR to the minimum can significantly affect food quality [17]. 

Nanoparticles have been widely used to improve performance of food packaging materials. TiO_2_ plays a major role in novel food packaging materials since it has high photosensitivity [18], oxidizability, long-term stability, and, last but not least, it significantly improves the antibacterial performance of materials [19]. It has been approved by the American Food and Drug Administration for use in materials that come into contact with food, such as food packaging. The incorporation of TiO_2_ nanoparticles may result in suitable food packaging materials, since those nanoparticles act as bioactive compounds that are key to the improved performance and added value of viable materials. 

This research presents a new, robust, but straightforward one-pot method for designing and synthesizing biodegradable, highly mechanical ethyl cellulose films. The design of such materials was inspired by the preparation of ethyl cellulose gels, where ethyl cellulose was first dissolved in ethanol and formed a gel with the addition of water [20]. An antisolvent caused the precipitation of ethyl cellulose from ethanol, creating a ‘paper-like’ structure. Since the solubility of ethyl cellulose in ethanol is limited at room temperature, the temperature of sol in this research was increased to 70 °C, resulting in a slightly yellow, highly viscous solution. At this temperature, concentrations up to 30% were achieved. The preparation method is adapted from Lim et al. [21], briefly, by casting the prepared solution onto a Petri dish and allowing the solvent to evaporate for film formation. Due to the simplicity of the process, the materials are highly reproducible and applicable in various fields. 

## 2. Materials and Methods

### 2.1. Synthesis and Characterization of TiO_2_ Nanoparticles

The TiO_2_ nanoparticles were prepared by the controlled hydrolysis of TiCl_4_. Briefly, 6 mL of TiCl_4_ (−20 °C) was added to 200 mL of deionized water at 4 °C while under stirring and left at this temperature for 30 min. Slow growth of the particles was achieved by dialysis against water at 4 °C until a pH of 3.5 was reached. The obtained colloidal solution was dried at 100 °C to evaporate the water and obtain TiO_2_ nanoparticles. Zeta potential was determined by dynamic light scattering (DLS, Zetasizer Nano, ZS, Malvern, UK) using TiO_2_ ethanol dispersions at 0.1 g·L^−1^. 

### 2.2. Formation of Ethylcellulose Film

The ethyl cellulose (48.7% *w*/*w* ethoxyl basis; degree of substitution: 2.5; viscosity: 57 MPas) used in the experiments was purchased from Sigma-Aldrich (St. Louisu, Missouri, USA). Ethyl cellulose films were synthesized by adding a specified amount of polysaccharide into preheated ethanol (absolute anhydrous; Carlo Erba Reagents, Val de Reuil, France) or ethyl lactate (70 °C) ((S)-(-)-ethyl lactate for synthesis; Merck KGaA, Darmstadt, Germany) while under stirring, and 10 wt%, 20 wt%, and 30 wt% solutions were prepared. The baker was sealed to prevent evaporation. After heating and stirring for several minutes, the ethyl cellulose powder was dissolved and a slightly yellow solution was obtained. The resulting solution was transferred into a Petri dish which was placed in a refrigerator at 6 °C to cool down. Once formed, the ethyl cellulose gel was left at 6 °C or placed at room temperature to completely dry. 

### 2.3. Formation of Ethyl Cellulose–TiO_2_ Film

Ethyl cellulose films with incorporated TiO_2_ nanoparticles were prepared, briefly, as described above in Section 2.2. In the initial step, 25 mg of TiO_2_ nanoparticles was first added to 20 g of ethanol and then heated to 70 °C. Ethyl cellulose was added to the TiO_2_–ethanol solution and a final 10 wt% solution was obtained. The further process was the same as described in Section 2.2. Samples of 10% EC (EtOH) TiO_2_ were prepared.

### 2.4. Film Thickness

The thickness of ethyl cellulose films was determined with a digital caliper (Mitutoyo, Andover, Hampshire, England & Wales (U.K.)). The sensitivity of the caliper was 0.01 mm. Results are reported as an average of three measurements.

### 2.5. Structural Analysis

Micrographs of the prepared films were obtained with a field emission scanning electron microscope (FE-SEM) Sirion 400 NC (FEI, Eindhoven, The Netherlands) equipped with an energy dispersive spectrometer (EDS). EC films were coated with gold particles and fixed to aluminum sample holders with double-sided carbon tape. Then, they were scanned at an accelerating voltage of 10–15 kV using a WD detector. SEM–EDS provides detailed information about the morphology and elemental composition of samples. 

### 2.6. Mechanical Analysis

The mechanical properties of EC films were evaluated by tensile testing using a universal testing machine (Shimadzu AG-X plus 10 kN). Samples were cut to 30 mm × 4 mm. The nominal tensile strain (*ε*) was defined by the Equation (1):

(1) $$\varepsilon =\frac{∆l}{l_{0}}·10$$

where Δ*l* is the change in the length and *l_0_* is the undeformed length of the specimen. The nominal stress (*σ*) was obtained by Equation (2):

(2) $$\sigma =\frac{F\prime }{A_{0}\prime }$$

where *F′* is the force and *A_0_′* is the original cross-sectional area of the specimen. The loading rate was 100 mm/min for tensile tests. 

### 2.7. Water Absorption Test

Films were cut into 1.5 cm × 1.5 cm samples and weighed (*W*_1_). Samples were then placed into deionized water at room temperature. Samples were collected at predetermined times; the surface was slightly blotted with tissue paper to remove excess water droplets. Samples were weighed again (*W*_2_). The test lasted for three months. Water absorption was then calculated by Equation (3):

(3) $$water\;absorption\;\left(\%\right)=\frac{W_{2}-W_{1}}{W_{1}}·100\%$$

For each sample, the test was repeated three times to determine the repeatability of the experiment.

### 2.8. Static Water Contact Angle

To estimate the wettability of the films, one droplet (10 μL) of ultrapure water was deposited on the ethyl cellulose film surface (1 × 1 cm^2^) with the electronic pipette. The static contact angle was obtained after the water drop reached equilibrium on the film surface. A magnified picture of the droplet was taken with a Nikon DSLR D750 + NIKKOR 24–70 mm and uploaded onto ImageJ software, where the contact angle was measured between the baseline of the drop and the tangent at the drop boundary, as described by Lamour et al. [22]. 

### 2.9. Thermal Properties

The thermal degradation of ethyl cellulose films was characterized using a thermogravimetric analyzer (TGA/DSC1, Mettler Toledo AG (MTANA), Zürich, Switzerland). The dried films (~ 20 mg) were placed on a ceramic pan and heated from 25 °C to 600 °C. The heating rate was 10 °C·min^−1^ using a furnace under a dry N_2_ purge of 50 mL·min^−1^. Differential scanning calorimetry (TGA/DSC1, Mettler Toledo AG (MTANA), Switzerland) was used to determine the glass transition (*T*_g_) and melting (*T*_m_) temperatures of the films. 

### 2.10. IR Spectroscopy

ATR-FTIR spectra of 10% EC (EtOH), 10% EC(EL), and 10%Ec (EtOH) TiO_2_ were recorded using an Schimadzu IRAffinity-1S FTIR spectrometer with an attenuated total reflection (ATR) module at a scan range of 4000–400 cm^−1^. The presented spectra are averages of 32 consecutive measurements. 

### 2.11. X-ray Diffraction

X-ray diffraction (XRD) patterns of the prepared material were recorded on a PANalytical X’Pert PRO high-resolution diffractometer with CuKα1 radiation in the 2θ region from 5° to 70° using a step of 0.034° per 100 s. 

### 2.12. Oxygen Transmission Rate

The oxygen transmission rate (OTR) was performed as described in the literature [23] on a PERME® OX2/230 (Labthink Instruments Co., Ltd., Jinan, China). Briefly, the testing area was 50 cm^2^, and oxygen flow was 10 mL/min. The relative humidity was 50%, the temperature was 23 °C, and EC films were conditioned for 24 h before testing. 

### 2.13. Water Vapor Transmission Rate

The EC films’ water vapor transmission rate (WVTR) was determined according to the literature [23] using the desiccant method. The film was cut into a circle of 6.3 cm diameter and sealed onto an aluminum permeation cup containing 45 g of dry calcium chloride with silicone grease and a ring to hold the film in place. Briefly, the climatically controlled chamber was set to 25 °C, and 65% RH and films were weighed at regular intervals. A linear relationship was obtained between the quantity of water transferred per unit of air and time. 

## 3. Results

**Figure 1:**
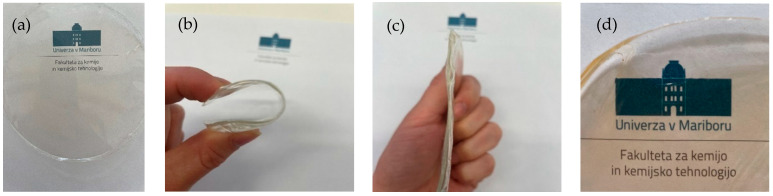
(**a**) EC film prepared in EtOH. (**b**) Bendable properties of the film. (**c**) Shape recovery after the forced removal. (**d**) TiO_2_-incorporated EC film prepared in EtOH.

In this research, ethyl cellulose (EC) films were prepared in EtOH (10, 20 and 30%) or EL (10%). First, the EC was dissolved in the solvent at 70 °C and then cooled down to 6 °C to form a film, which was further dried at 6 °C by the evaporation method. Ethanol and ethyl lactate were chosen as solvents based on the solubility of ethyl cellulose and based on their toxicity data. Completely transparent and flexible films were formed. As shown in Figure 1, the EC films exhibited extraordinary mechanical properties. The prepared samples were strong and did not show any visible damage after removing the deformation force, as shown in Figure 1. EC films quickly recovered to their initial shape. The incorporation of TiO_2_ nanoparticles turned completely transparent films slightly yellowish, clearly indicating the successful and homogeneous entrapment of nanoparticles in the EC films without clusters or nanoaggregates. Despite the yellow color of the TiO_2_-incorporated films, transparency was not lost (Figure 1d). The size of the synthesized nanoparticles was 114.3 ± 15.4 nm and their zeta potential was 26.9 ± 0.4 mV. The tensile strength and elastic modulus of the EC films dried at lower temperatures were compared to a sample prepared at room temperature (Table 1). These results are important with respect to food packaging applications of these materials. The sample prepared at room temperature with a standard preparation method described in the literature showed a high tensile strength and elastic modulus. This sample was less flexible than samples prepared at lower temperatures. The tensile strength of the EC films prepared at 6 °C decreased with decreasing EC concentration and was nearly the same for EtOH and EL, used as solvents. Similarly, the elastic modulus increased with increasing concentration of EC in the final film, and the most rigid film was observed to be the one with the highest EC concentration (30%). Room temperature is not favorable for obtaining materials with high tensile strength or low elastic modulus; therefore, it is clear that the use of plasticizers is desired. In addition, the incorporation of TiO_2_ nanoparticles into the 10% EC (EtOH) film did not drastically change the mechanical properties, since the tensile strength was nearly the same and an elastic modulus of 552 MPa was achieved. 

**Figure 2:**
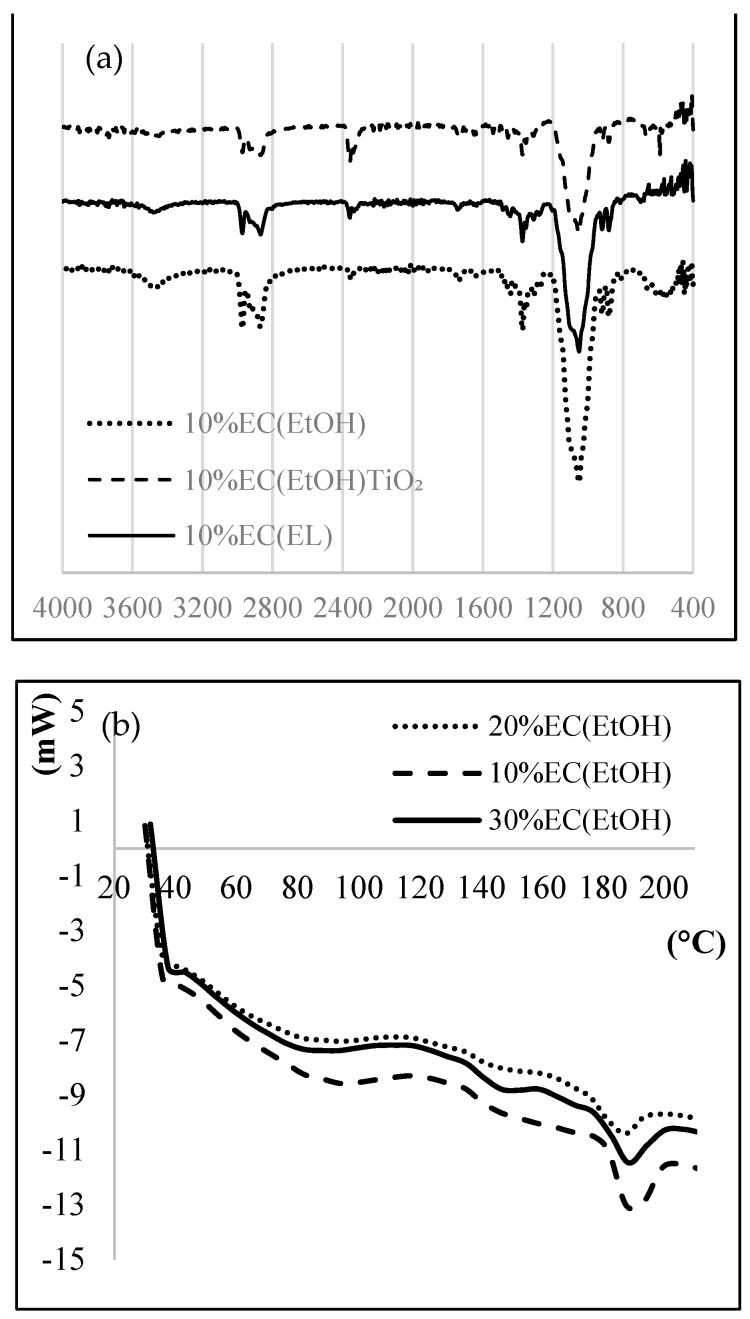

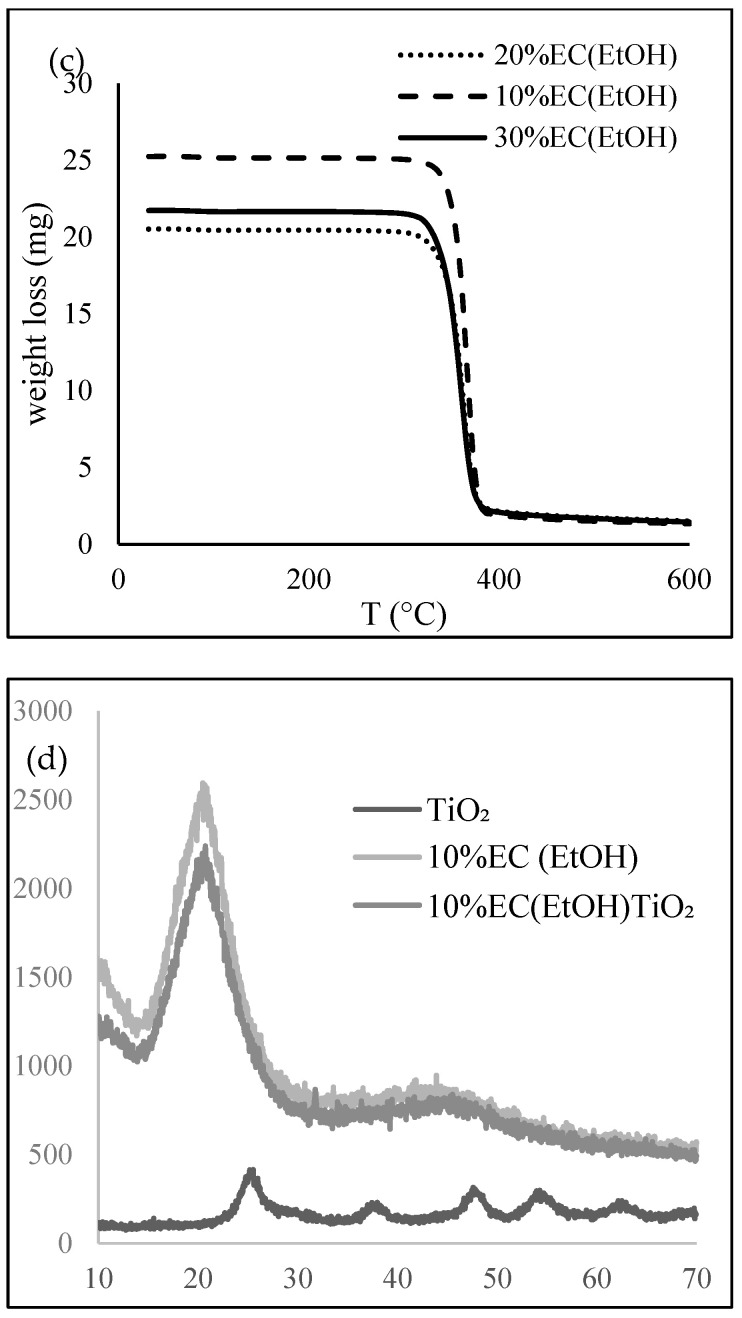
(**a**) FTIR, (**b**) DSC, (**c**) TG, and (**d**) XRD of prepared EC samples.

The IR spectrum of EC films (Figure 2a) showed a band at 3472 cm^−1^ attributed to OH stretching vibrations. Due to the C-H stretching vibration peak, FTIR of EC showed characteristic peaks at 2974 cm^−1^ and 2869 cm^−1^. The other essential peaks at 1091 and 1373 cm^−1^ corresponded to C-O-C stretching and C–H bending, respectively. The peak observed at 590 cm^−1^ was due to the vibration of the Ti-O-O bond. The FTIR spectrum firmly suggests the presence of Ti-O bonds.

Thermal analysis was performed to investigate the thermal behavior of the EC films. A DSC thermogram (Figure 2b) showed an exothermic peak at 188.4 °C, mainly due to the crystallization temperature of the sample, indicated for the amorphous form of the prepared films. EC films are hydrophobic and thus they do not retain any moisture; therefore, there is no mass loss near 100 °C. The TG graph (Figure 2c) shows that the complete degradation of EC film occurred at 378.8 °C for all prepared samples. More than 90% total weight loss was observed after 380 °C. 

X-ray diffractograms (Figure 2d) of 10% EC (EtOH) and 10% EC(EtOH) TiO_2_ films showed broad peaks at 2θ equal to 20.46 and 20.42, respectively. The XRD analysis of TiO_2_ nanoparticles showed that all diffraction peaks in the spectrum were related to the characteristic peaks of the TiO_2_ anatase phase. The diffraction peaks appeard at 2θ = 25.18, 37.95, 47.59, 54.25, 54.69, and 62.14, and their corresponding tetragonal crystal planes were (101), (004), (200), (105), (211), and (204), respectively. 

**Figure 3:**
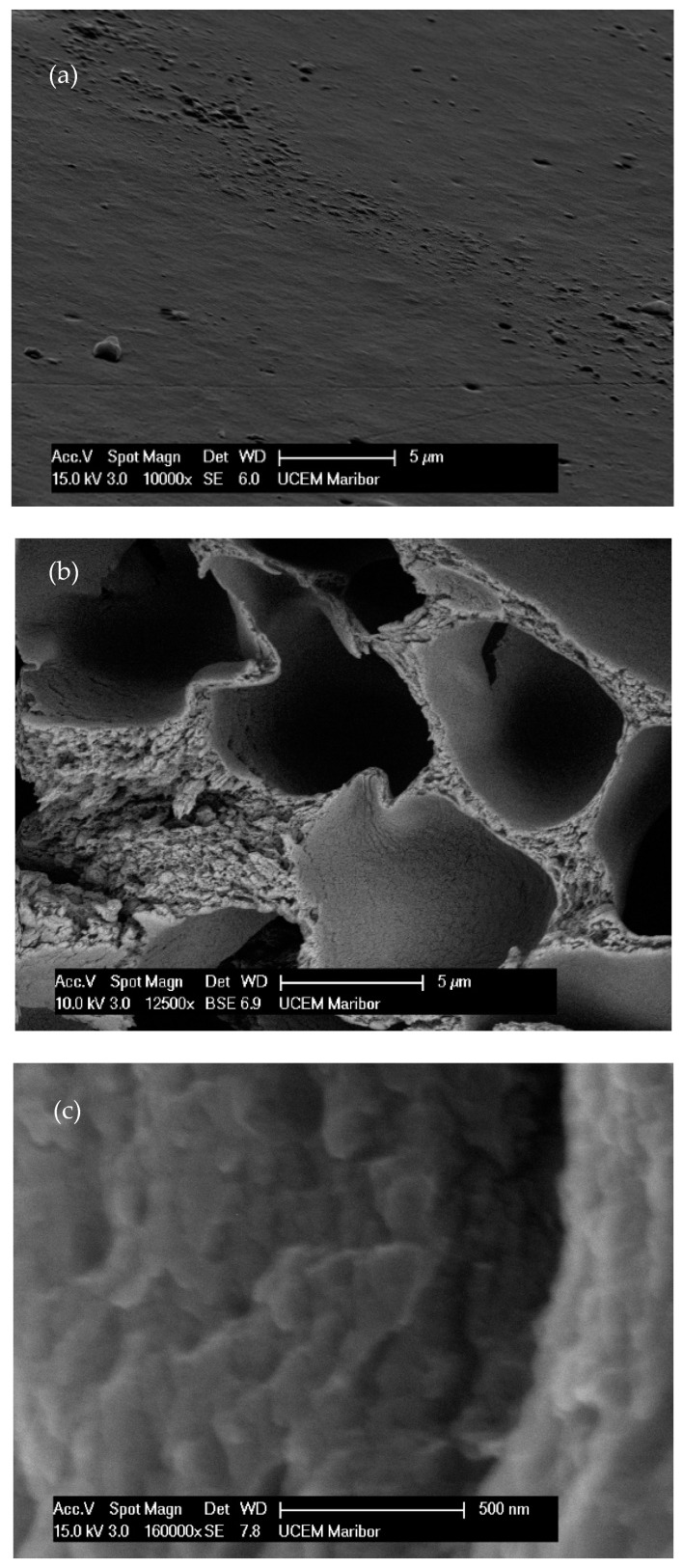

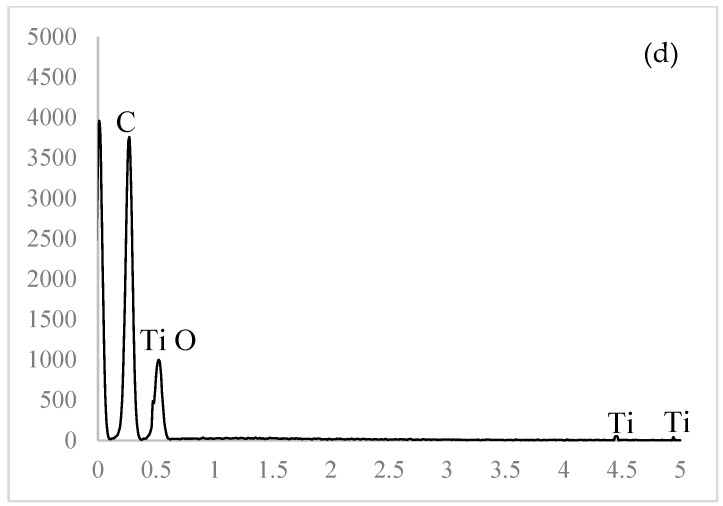
FE-SEM micrographs of the EC films. (**a**) Surface of film. (**b**) Cross-section of film. (**c**) TiO_2_ deposition inside EC film. (**d**) EDS analysis of the 10% EC(EtOH)TiO_2_ film.

The obtained micrographs of the prepared samples are shown in Figure 3. A smooth surface without pores, voids, or aggregates is visible in Figure 3a. The prepared samples had uniform and coherent surfaces with porous intersections. The inner structures had honeycomb-like porous structures with open and closed uniform macropores. The inner structures appeared highly organized. Walls, separating larger voids, were compact but porous. According to the SEM image, these pores were in the mesoporous range. The micrographs of the 10% EC(EtOH) TiO_2_ film are shown in Figure 3c, in which TiO_2_ nanoparticles are clearly visible and their sizes correspond to the zeta potential measurements. The EDS analysis was performed on the 10% EC(EtOH) TiO_2_ sample, showing the presence of Ti, O, and C. Since a low amount of TiO_2_ nanoparticles was used for the film preparation (25 mg), the weight% was 0.1, whereas the weights% of C and O were 61.98 and 37.92, respectively. The results of the elemental analysis are shown in Figure 3d. The Lα for Ti was 0.4522, and thus this peak may collide with C and O peaks; however, there was small peak generated at Kα 4.5089 corresponding to the small amount of Ti in the sample. 

**Figure 4:**
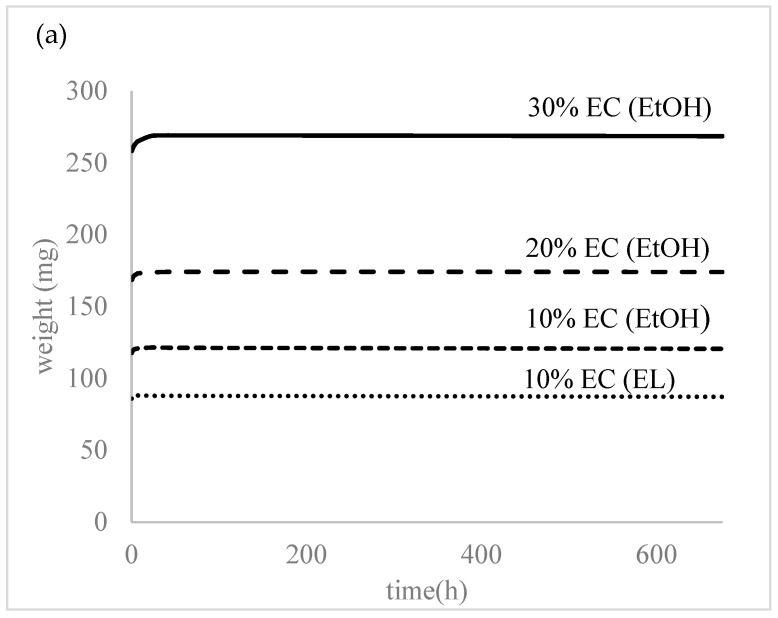

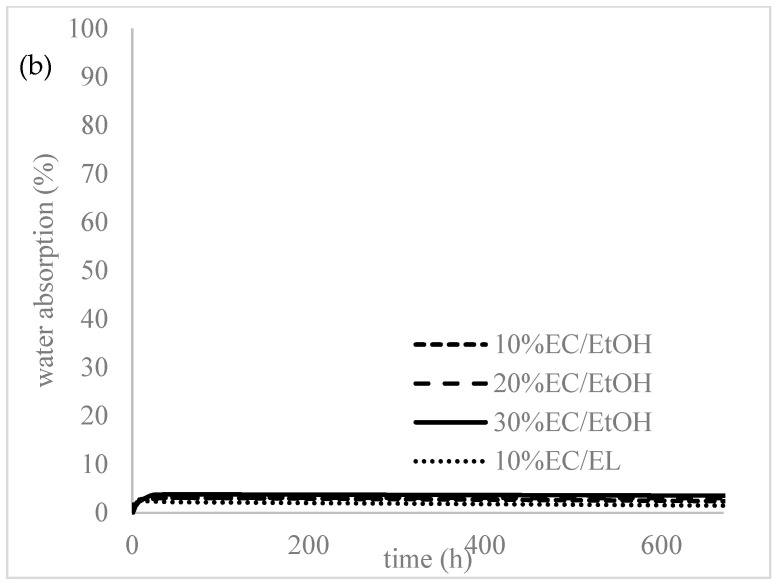
(**a**) Degradation and (**b**) water absorption of the prepared samples.

All the prepared samples were subjected to water to evaluate the swelling and degradation rate and water uptake. As seen in Figure 4, the prepared samples did not show any swelling or water absorption; the initial water uptake (2–3%) was probably due to water attachment on the surface or insufficient bloating of the samples. The samples were left in distilled water for over a month and there was no degradation or increased swelling observed at this time. 

**Figure 5:**
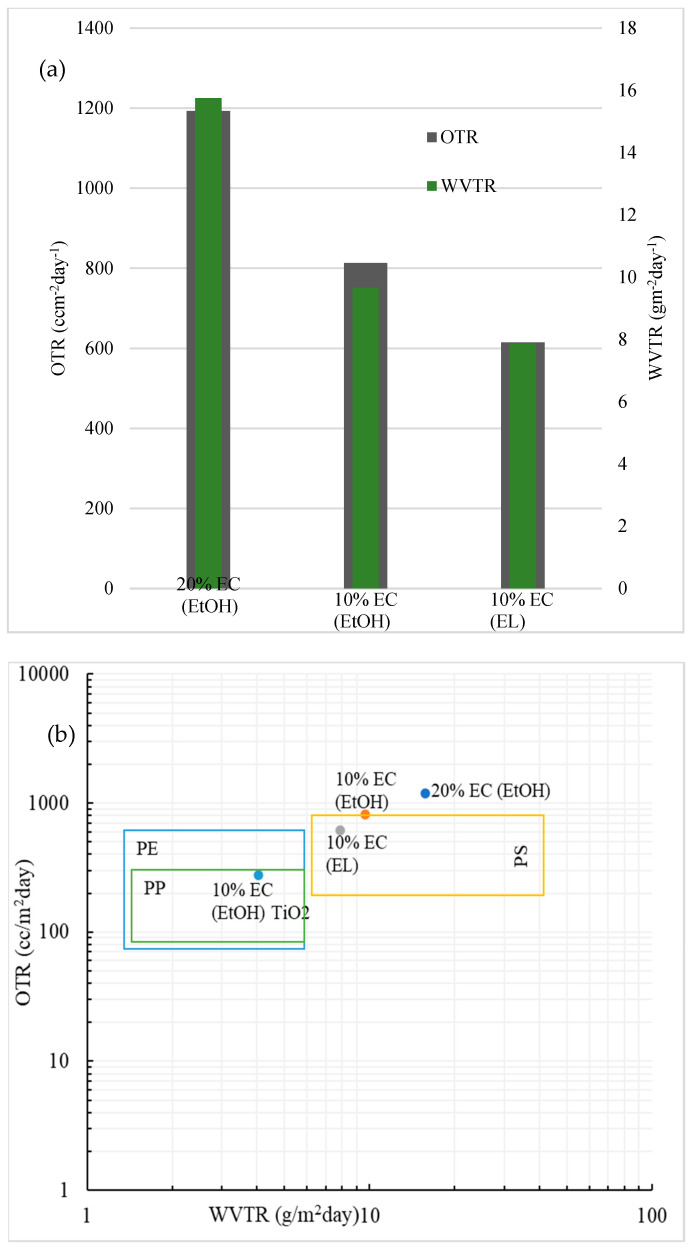
(**a**) Barrier performance of the prepared samples; normalized data for 60 μm-thick films. (**b**) Comparison of the barrier performance of the prepared EC films in this study with those of some common polymers used in food packaging. Data are normalized for 60 μm-thick films.

The OTR and WVTR results for prepared EC samples are shown in Figure 5a. The data are normalized for 60 µm-thick films to compare the results accurately. The WVTR values of all samples were low, which could be expected from the hydrophobicity and water uptake results and is in the range of some common plastic materials, as seen from Figure 5b. The WVTR value was below 10 g m^−2^day^−2^, except for the film with 20% EC. The values of the prepared EC films were compared to polystyrene (PS), polypropylene (PP), and polyethylene (PE) [24]. The OTR value for blank films was in the range of polystyrene [25]. The lowest OTR value for blank film was observed in the 10% EC (EL) sample, indicating that ethyl lactate (EL) could potentially be a better solvent for preparing flexible EC films than ethanol. Among the prepared blank films, the 10% EC (EL) had the lowest moisture permeability (5.7 × 10^−11^ g m/Pa s m^2^), which was much lower than some typical gelatin films used in food packaging nowadays [26]. The sample prepared with the incorporation of TiO_2_ nanoparticles provided the lowest OTR and WVTR values, in the range of polypropylene, as seen in Figure 5b. 

## 4. Discussion

A low evaporation temperature of 6 °C allows the formation of stable and transparent EC films with enhanced properties compared to EC films that are prepared at room temperature. Mechanical properties are improved in terms of higher elasticity, which could be due to the slower evaporation of the solvent in the preparation phase. The evaluation of mechanical properties is usually performed through the elastic modulus. If the elastic modulus is lower, this corresponds to materials with higher elasticity. Another indicator is tensile strength, with higher values indicating more robust materials. Table 1 shows that the E value increased with the EC content, i.e., the EC film was less flexible when more EC was used in the preparation process. The highest E value was observed for the sample prepared at room temperature, clearly indicating that drying temperature strongly affects the final mechanical properties of EC films. This result suggests that the samples prepared at lower temperatures were more flexible and elastic and have properties similar to those with added plasticizers [27]. The elastic modulus of 10% EC in EtOH reported in the literature was between 500 and 1500 MPa [27], depending on the plasticizer used, which is in the range of our results obtained without plasticizers. This research shows that such low values are obtained only by lowering the temperature in the preparation process from room temperature to 6 °C (Table 1). In addition, besides the simplicity of such a method described in this research, this process represents an advance with respect to the transparency of final EC films. With the plasticizers used, the EC films turned white. This simple process, described first in this research, thus allows the preparation of flexible and bendable films without compromising their transparencies.

The lowest elastic modulus was achieved with 10% EC films prepared in ethanol and ethyl lactate, which could also be correlated with this film’s having the lowest EC concentration and thickness. However, the thickness of the reference film 10% EC (EtOH) T_room_ was nearly the same as the 20% EC (EtOH), though the elastic modulus was much higher. The tensile strength increased with the higher concentration of EC in the final film, but the highest tensile strength was achieved with the EC film dried at room temperature, which showed the lowest elasticity. This could be due to the EC molecules being closely packed together at higher concentrations [28]. The incorporation of TiO_2_ nanoparticles did not affect the film’s mechanical properties. By determining the contact angle, we showed that the materials are hydrophobic (Table 1), with the highest value observed for the 30% EC film prepared in ethanol. The contact angle decreased for less concentrated films, except for the film composed with ethyl lactate. This 10% EC (EL) film exhibited a contact angle above 95°—significantly higher than the same film prepared in ethanol (10% EC (EtOH)). Since the contact angle is directly associated with the number of polar surface functional groups [29], it is assumed that there are more polar functional groups with ethanol during the dissolution of ethyl cellulose than ethyl lactate. Since the contact angle of the sample prepared at room temperature was slightly higher than the sample prepared at 6 °C, it is assumed that the formation of inter-particulate bonds between ethyl cellulose molecules is based on temperature and that there were more polar groups at the film surface after room-temperature drying. The incorporation of TiO_2_ nanoparticles resulted in a much higher contact angle compared to the pure 10% EC(EtOH) films. The contact angle with nanoparticles incorporated in the film was the highest among the samples. Hydrophobicity was confirmed by the water uptake test, where the values were less than 4% of the total weight of all prepared materials (Figure 3). There was no water uptake or degradation of the film after 30 days. This was additionally confirmed by the swelling experiment, which showed that the samples did not swell or degrade in the presence of water after more than one month. The initial water uptake of less than 10% can probably be attributed to insufficient bloating. The presence of water does not affect the prepared materials, indicating that the degradation of those materials could not be observed in the presence of water. However, the rapid degradation of the prepared materials was observed in ethanol. The DSC and TG data show that the samples’ thermal behavior is the same as for raw EC and that the concentrations of the prepared films do not affect their thermal properties. The degradation of all samples occurs at around 380 °C. FTIR analysis showed that the EC did not interact with the solvent (EtOH or EL) since the significant peaks were the same. The stretching vibration of Ti-O bonds proved the presence of TiO_2_ in the sample. The EDS analysis confirmed the presence of TiO_2_ nanoparticles in the 10% EC(EtOH) TiO_2_ sample and confirmed the absence of other elements. SEM images of this sample also confirmed the size of nanoparticles as provided by zeta potential measurements. All the diffraction peaks of TiO_2_ were characteristic peaks of TiO_2_ in the anatase phase. Broad peaks of EC in the prepared films showed on the amorphous nature of prepared films, and the absence of TiO_2_ peaks in the 10% EC(EtOH) TiO_2_ film may be due to the small amount of TiO_2_ incorporated into the film or the collision with EC peaks. 

The WVTR and OTR values of the film are two critical parameters for evaluating the performance of materials as barrier packaging. We showed that the values of the prepared films were in the range of new bioplastic materials and even in the range of some common materials, such as polystyrene [25]. The incorporation of TiO_2_ nanoparticles shows excellent potential in this type of application. It is reported that TiO_2_ nanoparticles act as antimicrobial agents and thus significantly improve the antibacterial performance of final materials [19]. The incorporation of TiO_2_ affects barrier properties and gas transmission. The lowest OTR values were obtained with the 10% EC film prepared in ethanol with the incorporation of TiO_2_. This may have been the result of oxygen absorption by TiO_2_, as reported elsewhere [18]. Since the absorption of oxygen is significantly higher than for pure EC films, the transmission rate is expected to be lower. Since incorporating TiO_2_ nanoparticles is highly beneficial for the gas and water transmission rate and, as reported recently, has impressive antibacterial properties [30,31,32], this simple process of preparing EC films with incorporated nanoparticles shows high potential in active food packaging.

## 5. Conclusions

This research used a robust and straightforward method to prepare EC films with good mechanical properties without compromising transparency. EC films were prepared using the solution casting method and doped with TiO_2_ nanoparticles without plasticizers. Ethyl cellulose was dissolved at 70 °C in either ethanol or ethyl lactate. The obtained solution was set to cool down to 6 °C to form a film, then dried at 6 °C. This cooling and drying step was shown to be crucial for the mechanical properties of the EC films which later developed. Decreasing the temperature in the preparation step decreased the elastic modulus values compared to EC films with plasticizers incorporated. By this simple casting method, highly flexible EC films were obtained without compromising the transparency of the films. The thermal properties of the prepared films were the same as for pure EC; the degradation of the material occurred after around 380 °C and was not affected by the EC concentration. Micrographs of the prepared materials showed smooth, uniform, and nonporous surfaces, with the macroporous honeycomb-like intersections. Determination of the contact angles confirmed the hydrophobicity of all the prepared samples, which was also confirmed by the water uptake and swelling studies. These studies showed that water uptake was neglectable, and there was no degradation of EC films after more than a month spent in contact with water. By dissolving EC at higher temperatures, samples with up to 30% wt EC were obtained. The samples with the highest concentrations were the thickest (850 µm) and had higher elastic moduli and lower oxygen transmission rates than 10% EC samples. Samples prepared from 10% EC in EL showed the lowest elastic modulus but the highest WVTR and OTR values. The OTR and WVTR values were in the range of polystyrene and polypropylene; the sample observed to have the lowest values was that which incorporated TiO_2_ nanoparticles. The described process thus allows the uniform incorporation of TiO_2_ nanoparticles that may act as antimicrobial agents to improve antibacterial properties, allowing the production of materials suitable for the preparation of active food packaging materials, and lower OTR and WVTR values. The ethyl cellulose solution casting method at lower temperatures with deposition of TiO_2_ nanoparticles may present a simple yet straightforward and cost-effective method for the generation of new smart food packaging materials. 

## Figures and Tables

**Table 1:** Properties of the EC films.

Sample	Tensile Strength (MPa)	Elastic Modulus (MPa)	Thickness (mm)	Contact Angle (°)
30% EC (EtOH)	22.8	691.5	0.85	106.3
20% EC (EtOH)	18.0	625.8	0.45	84.5
10% EC (EtOH)	14.2	576.5	0.19	80.3
10% EC (EL)	14.3	528.0	0.13	102.6
10% EC (EtOH) T_room_	28.9	913.0	0.48	93.7
10% EC (EtOH) TiO_2_	14.5	552.3	0.15	107.6

## Data Availability

Not applicable.
